# MIMR: Development of a Web-Based System for miRNA and mRNA Integrated Analysis

**DOI:** 10.3390/ijms252111819

**Published:** 2024-11-04

**Authors:** Dayeon Kim, Younhee Ko

**Affiliations:** Division of Biomedical Engineering, Hankuk University of Foreign Studies, Yongin 17035, Gyeonggi-do, Republic of Korea; dayeonkim@hufs.ac.kr

**Keywords:** miRNA, mRNA, RWR, PPI network, pathway enrichment

## Abstract

The human body is a complex network of systems that is harmonized with multiple biological components. To understand these interactions is very challenging. With rapid development of advanced sequencing technologies, massive amounts of data such as mRNA, miRNA are rapidly accumulated. The integrated analysis of mRNA–miRNA has brought an extensive understanding of complex biological systems and pathological mechanisms. MicroRNAs (miRNAs) are small non-coding RNAs that intricately regulate target gene products, resulting in the inhibition of gene expression. While these miRNAs play crucial roles in essential biological processes—ranging from immunity and metabolism to cell death—their specific impacts on diseases remain unknown. Recent studies have been focused on the integration of miRNA and mRNA expression to reveal the underlying biological pathways and mechanisms responsible for disease manifestation. We proposed a novel approach for integrative analysis of miRNA and mRNA expression data and developed MIMR (Integrative Analysis of miRNA and mRNA), a web-based application that leverages the Random Walk with Restart (RWR) algorithm. MIMR incorporates both direct and indirect interactions by utilizing protein–protein interaction (PPI) networks and experimentally validated mRNA–miRNA target interactions. MIMR provides comprehensive results, including novel pathological pathways associated with a specific disease and interactive network diagrams representing the mRNAs and miRNAs. We applied it to Alzheimer and breast cancer data and successfully identified the novel biological pathways related to these diseases. In summary, MIMR will offer a deeper insight into the hidden mechanisms of diseases and identify potential therapeutic strategies through integrated analysis of miRNAs and mRNAs.

## 1. Introduction

The human body relies on complex interactions between various biological molecules, and malfunctions of these interactions lead to disease. MicroRNAs (miRNAs), small non-coding RNA molecules about 22 nucleotides in length, play a critical role in the body by binding to mRNAs and inhibiting their expression through degradation or translational repression [1]. These miRNAs are key regulators in essential biological processes, including the immune response, metabolism, and apoptosis [2]. Their dysfunction is closely associated with the development of numerous diseases, including cancer, cardiovascular disorders, and neurological conditions. For instance, the overexpression of miR-21 inhibits apoptosis in malignant cells, thereby contributing to cancer progression. miR-21 acts as an oncogene in several types of cancers, including glioblastoma multiforme, cervical cancer, and breast cancer [3]. Given their crucial roles, miRNAs have emerged as important biomarkers for understanding disease mechanisms and developing therapeutic strategies. Therefore, understanding these pathways is essential for developing effective therapeutic strategies.

Several web applications have been proposed to support miRNA research, but many have become unavailable (Table 1). Currently, available tools include miRNA and mRNA Integrated Analysis (MMIA) [4], miRGator [5], and MicroRNA Enrichment Analysis and Annotation (miEAA) [6]. MiRGator and miEAA only focus on differentially expressed miRNAs, while MMIA identifies differentially expressed genes with the consideration of the inverse expression relationship between miRNAs and their target mRNAs. However, these traditional methods are limited in considering the direct relationships between miRNAs and mRNAs, often overlooking indirect interactions associated with other biological molecules, which leads to incomplete understanding of disease-related biological networks.

To address these challenges, we propose a web-based application for functional analysis of disease-related miRNA and mRNA data called MIMR (miRNA and mRNA integrated analysis) at http://sysbio.hufs.ac.kr/MIMR accessed on 1 September 2024. MIMR employs the RWR algorithm [7] to incorporate miRNA target information with mRNA data, enabling the exploration of protein–protein interaction networks and the identification of hidden biological mechanisms. The RWR algorithm allows us to utilize the indirect interactions associated with miRNA targets and mRNAs, which help to uncover the potential disease etiology. To validate the effectiveness of MIMR, we applied it to Alzheimer’s disease (AD) and breast cancer data, demonstrating its potential to uncover hidden pathological mechanisms. MIMR was developed to maximize accessibility for biologists and offer a user-friendly interface and intuitive result visualization.

**Table 1:** List of methods for miRNA and mRNA integrated analysis.

Tool	Input/Output	Target Database	Features	Ref
MIMR	miRNA and mRNA list or both/miRNA–mRNA network, boosted genes, enriched pathways	miRTarBase, DIANA-TarBase, miRDB, TargetScan	Protein–protein interaction (PPI) network boosting with RWR algorithm based on validated and predicted database Direct and indirect interactions	
MMIA	miRNA and mRNA expression data/diseases, TF binding sites, and pathways	TargetScan, PicTar, PITA	Fisher’s exact test based on inverse expression patterns between miRNA and mRNA using predicted database Direct interactions	[4]
miRGator 3.0	A single miRNA/targets, expression data	miRecords, miRTarBase, DIANA-TarBase, miRDB, etc.	Fisher’s exact test based on inverse expression patterns between miRNA and mRNA using validated and predicted database No enriched pathway	[5]
miEAA 3.0	miRNA list/pathway, diseases, TFs, and drugs	miRTarBase	Use one validated database only Provide miRNA information based on direct interactions	[6]
miRNet 2.0	miRNA list or expression/targets, TFs, disease, and pathways	miRTarBase, TarBase, miRecords	Union-based approach based on validated and predicted database No filtering for miRNA targets Direct interactions	[8]

## 2. Results

**Figure 1:**
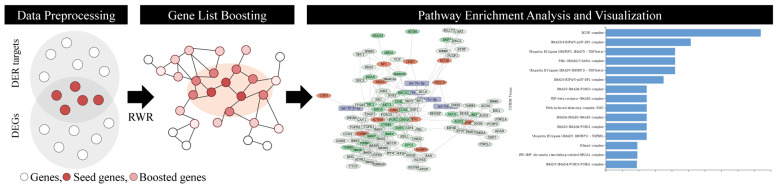
MIMR workflow comprises three steps: (1) data preprocessing, (2) gene boosting over a PPI network using RWR algorithm, and (3) pathway analysis. First, with lists of DER targets and DEGs, we identify their intersection with the seed genes for the RWR algorithm. In the second step, the RWR algorithm is applied over the PPI network to identify indirect hidden mechanisms associated with seed genes. Then, these boosted gene sets are used to discover hidden enriched pathways.

We developed MIMR, a web-based system for integrated functional analysis of miRNAs and mRNAs based on the RWR algorithm (Figure 1). It receives a list of differentially expressed miRNA (DER) and differentially expressed genes (DEG) from a user. The following three steps are processed: (1) data preprocessing, (2) gene list boosting and RWR propagation, and (3) pathway enrichment analysis and visualization.

We standardize miRNA names with precursor annotations designated as “−5p” and “−3p” and converted gene symbols to Entrez IDs. Then, given the compiled miRNA target databases, the targets of differentially expressed miRNAs (i.e., DERs) are imported into the seed gene list in addition to the DEGs. We provide various miRNA–target interaction databases (e.g., DIANA-TarBase, miRTarBase, TargetScan and miRDB). For RWR propagation, PPI network is constructed based on the STRING database. With our seed gene list (i.e., DEGs and targets of DERs), the RWR algorithm is applied to calculate the degree of association over the PPI network; then, the seed gene list is extended into the boosted gene list, leveraging the hidden indirect interactions between genes. With the boosted gene list, MIMR could identify such hidden etiological mechanisms indirectly associated with a disease condition. This boosted gene list could reveal critical biological mechanisms associated with pathological conditions causing diseases. MIMR allows users to explore biological networks associated with boosted genes and explore the enrichment result through various visualizations. The restart probability for RWR and boosting probability deciding the degree of propagation can be chosen. Finally, pathway enrichment based on both the seed and boosted gene list is provided with a network structure.

### 2.1. Case Study for Alzheimer Disease

**Figure 2:**
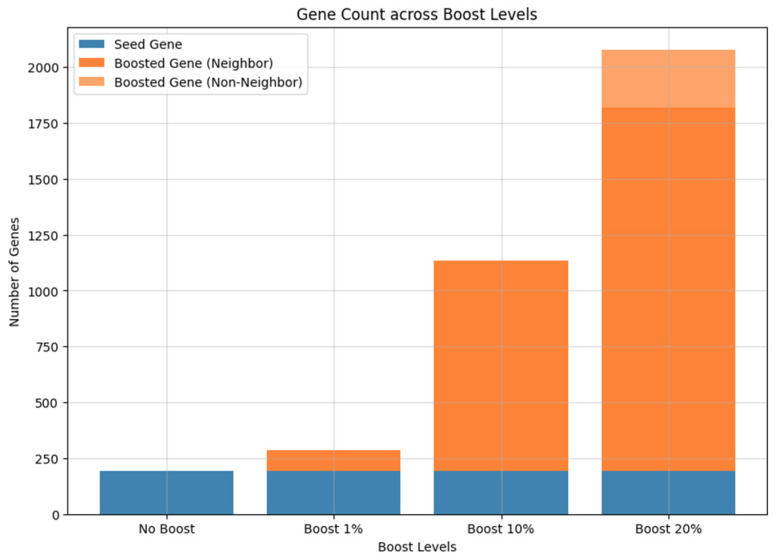
Distribution of the number of genes across different boosting levels with AD data. The *X*-axis represents the boosting levels (No Boost, Boost 1%, Boost 10%, Boost 20%), while the *Y*-axis shows the number of genes. The gene groups are categorized as seed genes (blue), boosted genes where they are directly/indirectly connected genes to seed genes (darker orange: 94, 939, and 1627 directly connected genes, lighter orange: 0, 3, and 257 indirectly connected genes).

**Figure 3:**
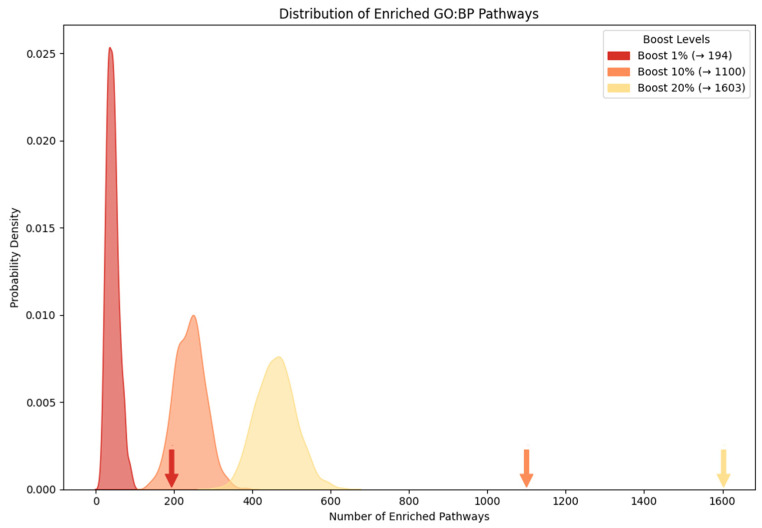
Expected distributions of enriched pathways across boosting levels (*p*-value < 0.01). The *X*-axis shows the null distribution of the number of enriched pathways based on different boosting levels (e.g., 1%, 10%, and 20%), and the *Y*-axis represents their probability density. Distributions are based on 10,000 random permutations of gene sets, using approximately 286 genes for 1% boosting (red), 1134 genes for 10% boosting (orange), and 2076 genes for 20% boosting (yellow). Arrows indicate the observed number of significant biological pathways at each boost level, all with a highly significant *p* = 9.999 × 10^−5^.

To demonstrate the effectiveness of our method in revealing novel biological pathways, we analyzed genomic data from Alzheimer’s disease (AD) patients. A total of 43 miRNAs are compiled from the prior study [9], where they are reported as differentially expressed in AD, and we also extracted 4239 common targets across two validated miRNA target databases (e.g., DIANA-TarBase and miRTarBase). Simultaneously, we identified 607 genes differentially expressed from all five experiments (GSE33000, GSE36980, GSE48350, GSE5281 and GSE122063) [10]. Among them, we selected 192 genes, representing the intersection of the target of DERs and DEG as seed genes. These gene sets are categorized as follows: the ‘seed gene set’ (192 genes), and the ‘boosted gene set’ at 1% (286 genes), 10% (1134 genes), and 20% (2076 genes) (Table S1, Figure S1). The boosted genes are defined as the high-scoring genes identified by RWR, which include seed genes. As expected, most of these boosted genes directly or indirectly interact with the seed genes (Figure 2). These findings indicate that the boosted genes naturally reflect the effect of hidden biological mechanisms to identify the etiology of disease (Table S2, Figure S2). The pathway enrichment analysis is performed with these boosted genes. As expected, the more boosted genes are considered, the more enriched pathways are identified (Figure 3). The significantly large number of enriched pathways reveals hidden biological associations of those boosted genes with a disease. For example, while the seed gene set primarily could highlight general terms ‘response to stress’, the boosted genes identify more specific pathways, such as ‘oxidative stress’, ‘stress-activated MAPK cascade’, and ‘stress fiber assembly’, all of which help to understand the pathology of AD (Table 2). Our analysis with boosted genes successfully identifies novel biological pathways associated with AD.

**Figure 4:**
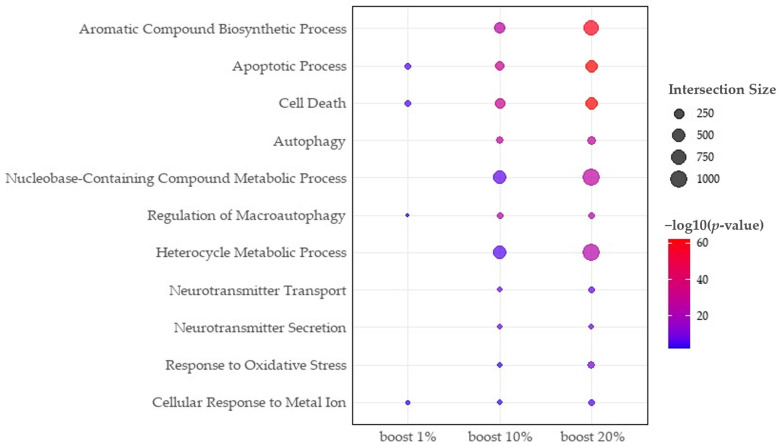
Dot plot for enriched pathway across boosting levels. The *X*-axis shows the boosting levels, and the *Y*-axis lists the representative enriched GO terms. The dot size indicates the number of genes associated with each pathway (intersection size), while the dot color represents enrichment significance, with darker red indicating higher significance.

We conducted a literature review based on enriched pathways with the boosted gene list (Table 3, Figure 4). Interestingly, our analysis identified critical GO terms closely related to AD, such as the apoptotic process [11], cell death [11], and regulation of autophagy [12], that could not be detected with the seed genes. Beta-amyloid aggregation, a major symptom of AD, triggers a significant cascade of neuronal cell death, and our boosted gene list successfully detected the enrichment of the apoptotic process and cell death pathway. Additionally, the nucleobase-containing compound biosynthetic process [13], heterocycle biosynthetic process [14], and aromatic compound biosynthetic process [15], are also enriched only in the boosted genes. The synthesis of heterocyclic compounds is crucial for maintaining neuronal function and is associated with purine and pyrimidine metabolism, both of which are disrupted in AD patients. Notably, metal ions [16], oxidative stress [16], and neurotransmitters [16] are also detected from our boosted gene list, which are closely associated with dysfunction of neurons. Oxidative stress is known to be a significant pathological factor in AD, as reactive oxygen species (ROS) damage neurons and promote beta-amyloid accumulation [16]. Tryptophan metabolism supports cognitive function and suppresses inflammation through neurotransmitter production [16]. Our PPI network constructed from seed genes and boosted genes revealed three biological modules associated with three hub genes: *SMAD3*, *HRAS*, and *MAPK8*. These hub genes show high degree values of 39, 38, and 33 (note that the average degree of node is 11.93), respectively, and eigenvector centrality scores of 0.21, 0.26, and 0.20 (i.e., the average centrality is 0.0055). Although *SMAD3* is not identified as a DEG or DER target, our network boosting with RWR successfully detected the *SMAD3* as a critical disease causative gene in AD. The activation of *SMAD3* signaling impairs macrophages’ ability to clear amyloid beta (Aβ), thereby promoting AD progression [17]. Through our network analysis, we also identified the closely interacted genes with *SMAD3*, such as *YAP1*, *SMAD4*, *ACVR2A*, and *ERBIN*. These genes are both DEGs and DER targets, regulated with miRNAs, including hsa-miR-26b-5p, hsa-miR-93-5p, hsa-miR-34a-5p, hsa-miR-15b-5p, hsa-miR-301a-3p, hsa-miR-146a-5p, hsa-miR-23a-3p, and hsa-miR-483-3p, which would be novel potential candidate miRNAs for AD. The identification of these hidden relationships associated with *SMAD3* shows the promising direction of our method.

### 2.2. Case Study for Breast Cancer

**Figure 5:**
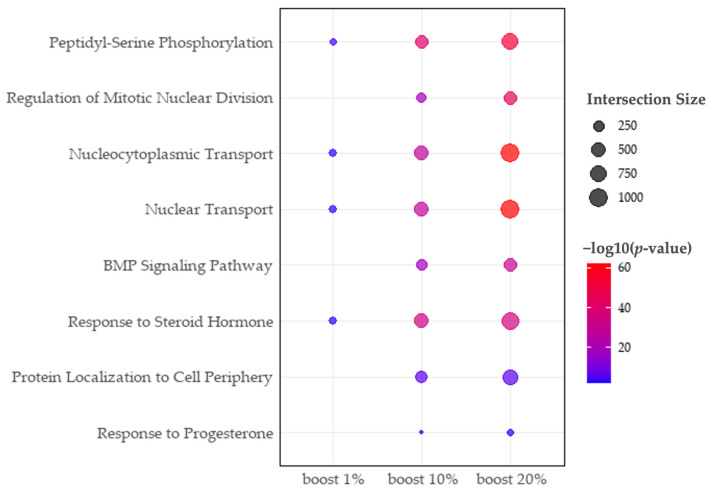
Dot plot of the enriched pathway across different boosting levels. The *X*-axis shows the boosting levels, and the *Y*-axis lists the representative GO terms. The dot size indicates the number of genes associated with each pathway (intersection size), while the dot color represents enrichment significance, with darker red indicating higher significance.

**Figure 6:**
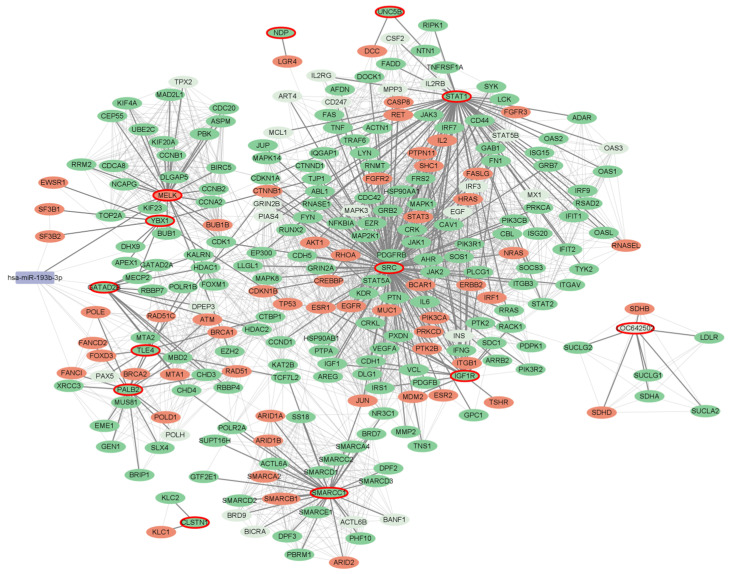
Expanded subnetwork of “Response to Progesterone” with 10% boosting level in BC. This network comprises miRNA (lavender rounded rectangles) and gene (ellipses) nodes, representing miRNA–target and protein–protein interactions. Seed genes are shown in orange, while boosted genes appear in green. Boosted genes, also known as DER targets or DEGs, are displayed in a darker green.

We applied our method to breast cancer (BC) data, using known miRNAs and causative genes. We compiled the 1820 miRNAs from RNADisease [18] and 769 mRNAs from GeneCards [19], resulting in 272 seed genes. The RWR algorithm is applied to the seed genes with a 10% boosting level, leading to the 1205 seed genes (Tables S3 and S4, Figures S3 and S4). Most of these boosted genes (i.e., 767 out of 933) are targets of DERs or DEGs, while the remaining 166 genes are not directly connected to the seed genes in the PPI network. However, those 166 boosted genes reveal the hidden pathway to explain the etiology of breast cancer. For example, while the enrichment test for seed genes identified the association of the “response to estrogen” pathway with breast cancer, the boosted gene set revealed more specific enriched pathways such as the “intracellular estrogen receptor signaling pathway”, involving estrogen binding to its receptor and regulating gene expression and cellular activities crucial for breast cancer growth [20]. Similarly, “ERBB2 signaling” and “ERBB2-ERBB3 signaling” pathways were identified from the boosted gene set, where *HER2* and *HER3* heterodimer signaling is vital for *HER2*-positive breast cancer by enhancing cell survival and proliferation [21]. Furthermore, the boosted gene set identified novel biological pathways which could not be detected with seed genes such as “the response to progesterone” and “steroid hormone signaling” pathways (Figure 5). The response to progesterone is particularly important, as progesterone receptors are commonly expressed in breast cancer cells and influence tumor growth [22]. Steroid hormones, including estrogen and progesterone, play significant roles in breast cancer development and progression [20,22]. The boosted gene set not only confirms known pathways but also elucidates novel interactions for comprehensive understanding of cancer biology. The further analysis of the progesterone-related network revealed 13 hub genes (comprising 1 seed gene, 1 boosted gene, and 11 boosted target genes) and directly interacting genes (57 seed genes, 176 boosted target genes and 14 boosted genes). In this subnetwork, mir-193b was identified as a core regulator, which is sensitive to progesterone receptor status [23,24]. Our method successfully identifies the progesterone pathway and mir-193b, utilizing the boosting strategy over the PPI network (Figure 6).

## 3. Materials and Methods

For the integrated analysis of mRNA–miRNA, various heterogeneous data are collected and compiled, including a miRNA target database, PPI network, and pathway information.

### 3.1. Database of miRNA Targets

With the increased interest of miRNAs, many miRNA target databases have been developed in recent years [25,26,27,28]. These databases are broadly divided into two categories based on their sources: those obtained through experimental validation [25,26] and those predicted using computational prediction [27,28]. The former is trustable but could not cover broad interactions, while the latter may lead to many false positives. DIANA-TarBase [25] is a reference database of experimentally supported microRNA (miRNA) targets. It offers an extensive collection of experimentally validated interactions covering 422,893 miRNA–mRNA interactions with 1084 miRNAs and 422,893 genes (release v8). MiRTarBase [26], the second-largest collection of experimentally validated miRNA databases, is manually curated and analyzed over approximately 11,000 papers, ensuring high reliability despite lack of comprehensive coverage. It encompasses 380,640 miRNA–target interactions, involving 2600 miRNAs and 15,065 genes as of 2021. TargetScan [27] is a widely used database and contains 10,165,094 miRNA–target interactions involving 2606 miRNAs and 159,314 genes as of 2021. The miRDB [28] utilizes the MirTarget algorithm, employing Support Vector Machines (SVM) and integrating data from TargetScan, PicTar, miRanda, and non-canonical interactions. As of the 2019 release, 3,375,741 miRNA–target interactions comprise 2656 miRNAs and 50,185 genes. However, they identified computationally predicted potential interactions but may include many false positive interactions. In our system, we offer various options for selection of miRNA–target databases depending on user preference. For a conservative approach, the user can choose the intersection of experimentally validated databases such as DIANA-TarBase and miRTarBase. Meanwhile, users interested in identifying a broader range of candidates can choose predictive databases such as TargetScan or miRDB. In our systematic analysis, we used the common data shared between two experimentally validated databases, DIANA-TarBase and miRTarBase, resulting in a dataset with 41,433 miRNA–mRNA interactions consisting of 901 miRNAs and 10,927 mRNAs.

### 3.2. Protein–Protein Interaction Network

Protein–protein interaction data were obtained from the STRING [29] database, encompassing both direct physical interactions and indirect functional associations. STRING integrates heterogeneous data from various sources like KEGG, Reactome, and BioCyc and is curated by experts. Our study focused on the top 1% of interactions to maximize confidence in the dataset.

### 3.3. RWR Based on the PPI Network and Pathway Enrichment

**Figure 7:**
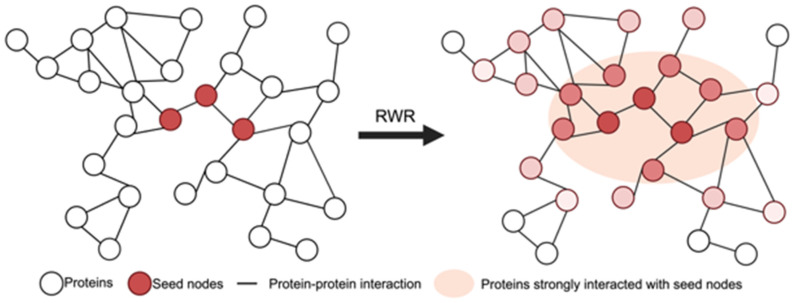
Extension of seed genes into the boosted genes by RWR. The seed nodes consist of genes of interest, such as DER target genes or DEGs. As the color of a node darkens, the distance score decreases, indicating a more robust interaction with seed nodes. This score serves as a cutoff threshold to select nodes having significant interactions with seed nodes.

Random Walk with Restart (RWR) is an algorithm that quantifies the closeness between nodes in the graph based on their connectivity [7,30]. Given a connected graph with transition probabilities, the RWR algorithm initiates from seed nodes and probabilistically explores the network by one of two actions: a random walk to an adjacent node or restart from the seed nodes. By iterating this process, the reaching probability of each node is calculated, thereby determining the distance score between the seed nodes and other nodes in the graph (Figure 7).

$$u_{new}=\left(1-p\right)Au_{old}+pu_{0}$$

where *p* is the restart probability and A is the adjacent matrix of the graph. $u_{0}$ denotes the initial probability distribution vector where only seed nodes are marked as 1, and $u_{new}$ represents the probability distribution vector where each element denotes the reachable probability. In our application of RWR, seed nodes are defined as the intersection of differentially expressed genes (i.e., DEGs) and target genes of differentially expressed miRNAs (i.e., DERs). We used the column-normalized adjacency matrix A from the STRING network. This approach is able to consider the systematic interactions between biological modules, which are often overlooked when focusing solely on DEGs or DERs. Since genes associated with the same etiological mechanisms tend to be clustered together in PPI networks, we incorporated this biological characteristic into the model using RWR for network exploration, as shown in Figure 7. Distance scores derived from RWR were used to identify genes that strongly interact with seed genes. These genes in the PPI network were then ranked according to their distance scores, and the top-ranked genes were extracted to generate a list of boosted genes, which are subsequently used for functional analysis to uncover hidden mechanisms associated with diseases. We applied the gene set enrichment test with g:Profiler [31], covering a range of biological databases to analyze functional pathways. In particular, comparisons of enriched pathways with boosted genes and seed genes are highlighted.

### 3.4. MIMR

MIMR was developed on the Apache HTTP server (version 2.4.18) under the Linux operating system. The server-side pipeline was written in bash scripts and Python. The RWR model was developed in C for enhanced speed. The client-side web interface utilized HTML5, JavaScript, and PHP (version 7.0.28), and jQuery (version 3.4.1), d3.js (version 4.0), Bootstrap (version 5.3.0), and Bootstrap-table (version 1.22.1) libraries were used. The bubble plot was implemented with plotly.js (v2.35.0).

## 4. Conclusions

We proposed an integrative approach for analyzing miRNA and mRNA data to provide a comprehensive understanding of disease mechanisms. To facilitate this, we developed MIMR, a user-friendly tool, which supports the integrated analysis of mRNA and miRNA. MIMR naturally incorporates the relationships between mRNA and miRNA into the analysis, which are often overlooked. The RWR strategy over a PPI network allows us to reflect indirect interactions which help to identify novel biological pathways or modules. A MIMR pipeline has proven effective in validating known miRNA and gene associations in diseases such as Alzheimer’s disease (AD) and breast cancer (BC). It efficiently discovers the potential disease-relevant genes based on seed genes through the boosting with a RWR algorithm. Our method will identify potential disease-associated mechanisms and novel therapeutic targets through the network boosting strategy.

## Figures and Tables

**Table 2:** Biological processes associated with “stress” in AD.

GO Term	Description	*p*-Value (Boost 1%)	*p*-Value (Boost 10%)	*p*-Value (Boost 20%)
GO:0006950	Response to stress	6.24 × 10^−10^	9.94 × 10^−58^	8.43 × 10^−79^
GO:0043149	Stress fiber assembly	N/A	4.28 × 10^−4^	7.95 × 10^−5^
GO:0051403	Stress-activated MAPK cascade	N/A	3.34 × 10^−4^	6.25 × 10^−5^

N/A: indicates non-significant findings.

**Table 3:** Enriched biological processes by 1%, 10% and 20% boosting set in AD.

GO Term	Description	*p*-Value (Boost 1%)	*p*-Value (Boost 10%)	*p*-Value (Boost 20%)
GO:0006915	Apoptotic process	1.69 × 10^−6^	7.04 × 10^−32^	6.95 × 10^−63^
GO:0008219	Cell death	2.69 × 10^−6^	1.69 × 10^−31^	5.03 × 10^−63^
GO:0016241	Regulation of macroautophagy	6.30 × 10^−3^	1.11 × 10^−23^	5.07 × 10^−25^
GO:0006914	Autophagy	N/A	2.52 × 10^−28^	6.31 × 10^−29^
GO:0006139	Nucleobase-containing compound metabolic process	N/A	9.31 × 10^−8^	1.08 × 10^−27^
GO:0046483	Heterocycle metabolic process	N/A	1.31 × 10^−6^	8.21 × 10^−25^
GO:0019438	Aromatic compound biosynthetic process	N/A	1.25 × 10^−26^	1.66 × 10^−59^
GO:0071248	Cellular response to metal ion	3.19 × 10^−4^	5.30 × 10^−4^	1.97 × 10^−4^
GO:0006979	Response to oxidative stress	N/A	4.28 × 10^−4^	1.32 × 10^−11^
GO:0006836	Neurotransmitter transport	N/A	1.26 × 10^−9^	1.83 × 10^−8^
GO:0007269	Neurotransmitter secretion	N/A	5.23 × 10^−9^	5.33 × 10^−9^

N/A: indicates non-significant findings.

## Data Availability

The original contributions presented in the study are included in the article/Supplementary Material. Further inquiries can be directed to the corresponding author/s.

## Supplementary Materials

The following supporting information can be downloaded at: https://www.mdpi.com/article/10.3390/ijms252111819/s1.
