# Supplementary material for: MIMR: Development of a Web-Based System for miRNA and mRNA Integrated Analysis
Source: Int J Mol Sci. 2024 Nov 4;25(21):11819. doi: 10.3390/ijms252111819 (PMC11546888; doi:10.3390/ijms252111819)
Supplement: Supplementary file 1 [file ijms-25-11819-s001.zip › ijms-3226262-supplementary.pdf]

Supplementary Material

Table S1. The number of genes by gene type in the 1%, 10%, and 20% boosting sets for AD. The table shows seed genes, target genes, only boosted genes, and the total for each set.

	seed gene	boost 1%	boost 10%	boost 20%
**seed gene**	192	192	192	192
**target gene**	0	35	377	722
**only boosted gene**	0	59	565	1162
**total**	192	286	1134	2076

Table S2. The number of genes by gene type in the 0.1%, 0.5%, and 1% boosting sets for AD. The table shows seed genes, target genes, only boosted genes, and the total for each set.

	seed gene	boost 0.1%	boost 0.5%	boost 1%
**seed gene**	192	192	192	192
**target gene**	0	4	16	35
**only boosted gene**	0	5	31	59
**total**	192	201	239	286

Table S3. The number of genes by gene type in the 1%, 10%, and 20% boosting sets for BC. The table shows seed genes, target genes, only boosted genes, and the total for each set.

	seed gene	boost 1%	boost 10%	boost 20%
**seed gene**	272	272	272	272
**target gene**	0	76	767	1480
**only boosted gene**	0	17	166	386
**total**	272	365	1205	2138

Table S4. The number of genes by gene type in the 0.1%, 0.5%, and 1% boosting sets for BC. The table shows seed genes, target genes, only boosted genes, and the total for each set.

	seed gene	boost 0.1%	boost 0.5%	boost 1%
**seed gene**	272	272	272	272
**target gene**	0	8	39	76
**only boosted gene**	0	1	7	17
**total**	272	281	318	365

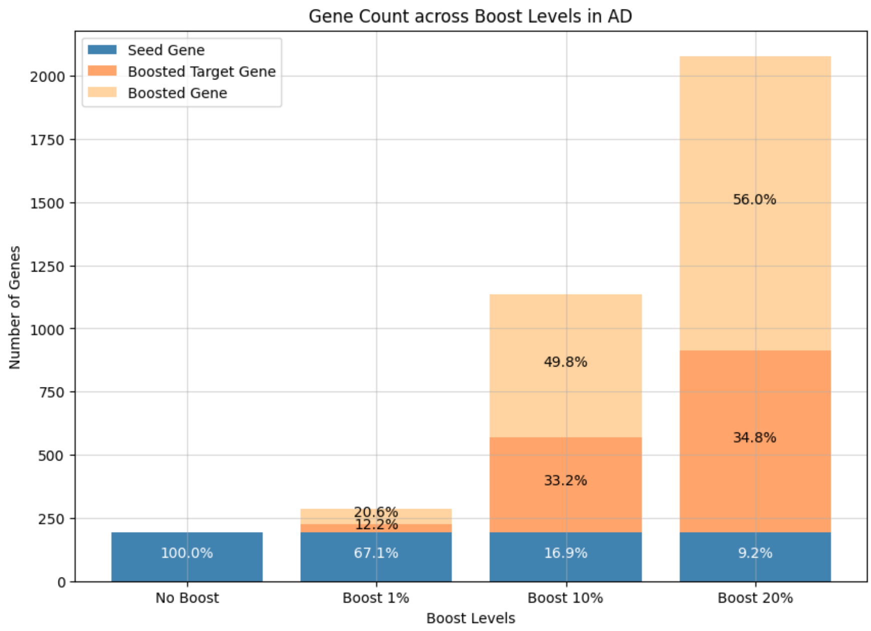

Figure S1. Stacked bar graph of the number of genes across different boosting levels with AD data. The X-axis represents the boosting levels (No Boost, Boost 1%, Boost 10%, Boost 20%), while the Y-axis shows the number of genes. The gene groups are categorized as seed genes (blue), boosted target gene (darker orange), and boost gene (lighter orange).

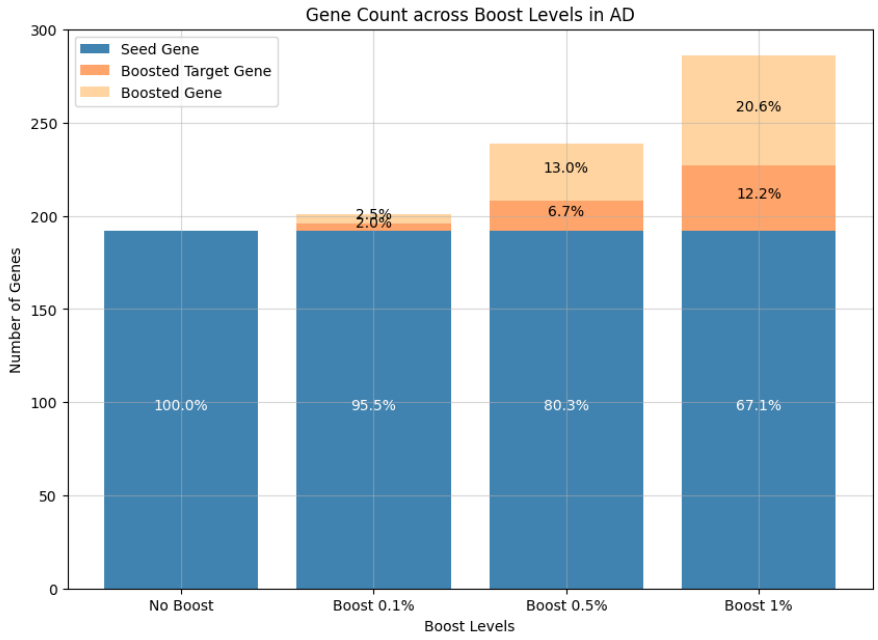

Figure S2. Stacked bar graph of the number of genes across different boosting levels with AD data. The X-axis represents the boosting levels (No Boost, Boost 0.1%, Boost 0.5%, Boost 1%), while the Y-axis shows the number of genes. The gene groups are categorized as seed genes (blue), boosted target gene (darker orange), and boost gene (lighter orange).

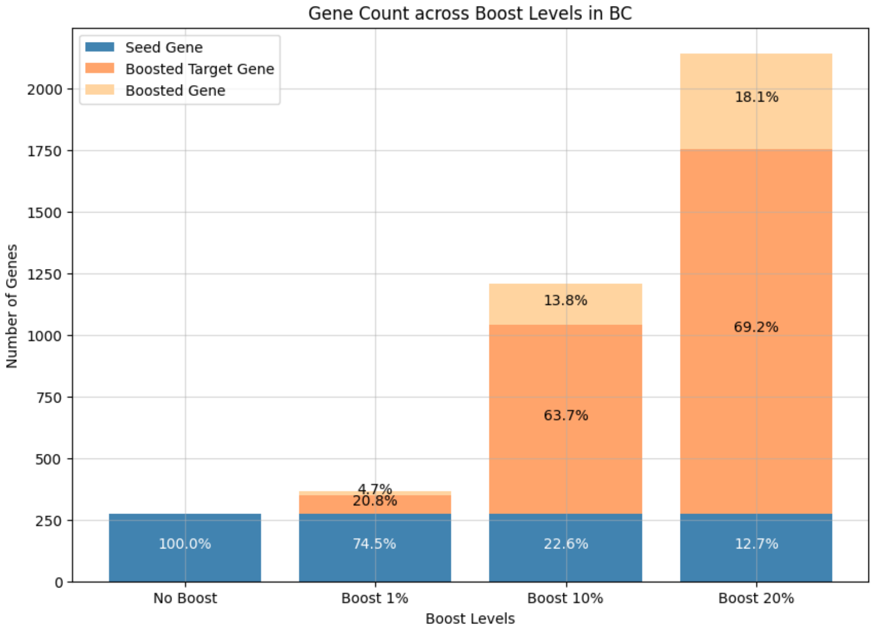

Figure S3. Stacked bar graph of the number of genes across different boosting levels with BC data. The X-axis represents the boosting levels (No Boost, Boost 1%, Boost 10%, Boost 20%), while the Y-axis shows the number of genes. The gene groups are categorized as seed genes (blue), boosted target gene (darker orange), and boost gene (lighter orange).

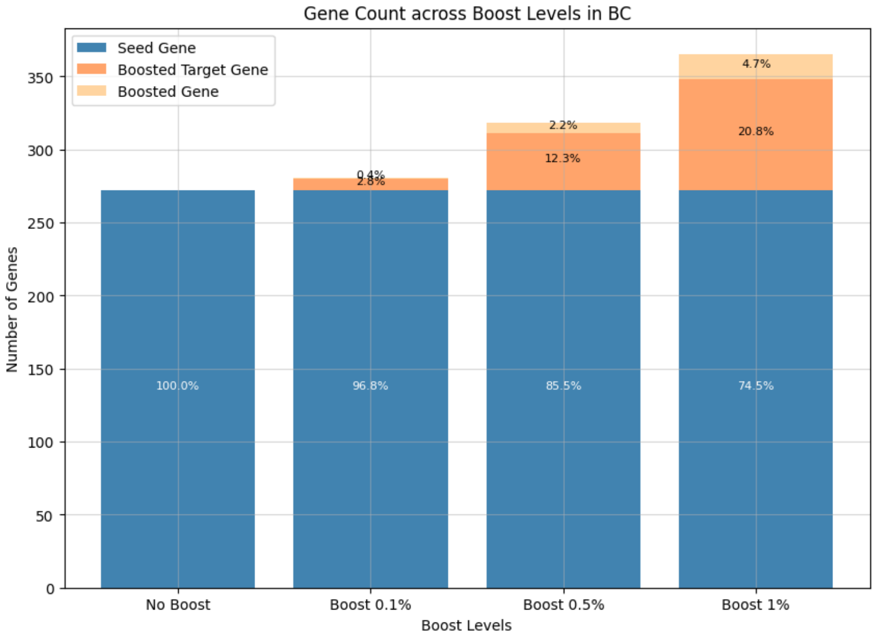

Figure S4. Stacked bar graph of the number of genes across different boosting levels with BC data. The X-axis represents the boosting levels (No Boost, Boost 0.1%, Boost 0.5%, Boost 1%), while the Y-axis shows the number of genes. The gene groups are categorized as seed genes (blue), boosted target gene (darker orange), and boost gene (lighter orange).
